# Residential care in California

**DOI:** 10.1080/17445647.2020.1768446

**Published:** 2020-06-05

**Authors:** Stephen Frochen, Seva Rodnyansky, Jennifer Ailshire

**Affiliations:** a Leonard Davis School of Gerontology, University of Southern California, Los Angeles, CA, USA; b Goldman School of Public Policy, University of California, Berkeley, CA, USA

**Keywords:** Residential care, older adults, geographical disparities

## Abstract

We examine the distribution of residential care in California, showing geographical disparities in care supply and need. We mapped the ratio of beds to older women in Los Angeles and San Diego County census tracts and concentrations of small and large facilities in the Cities of Los Angeles and San Diego. The largest ratios of residential care beds per older women occur on the border of the City of San Diego and on the periphery of Los Angeles County away from the City of Los Angeles. Clusters of small facilities take place in northern Los Angeles and southeastern San Diego, while clusters of large facilities occur in Downtown Los Angeles and near La Jolla. Understanding geographical disparities in residential care supply and need in California can help residential care developers, service providers, and local and state agencies partner in planning for residential care facility development in underserved areas.

## 1. Introduction

The older adult population in the United States is growing exponentially, estimated to nearly double to 80 million by 2050 (Ortman et al., 2014). As life expectancy increases in the US, so has the number of later life years spent in disease, comorbidity, and disability due to the long-term, chronic health conditions of old age, expanding the need for noninstitutional long-term care services and supports outside of the home (Crimmins et al., 2011). Because of this growing need for such support, the percentage of older adults residing in any form of senior housing is expected to more than double by midcentury (U.S. Department of HHS, 2003). As the largest senior housing type by the number of facilities, providing noninstitutional support for older adults who can no longer stay at home, residential care is an ideal outlet for this growing need. Typically more integrated into local communities than skilled nursing facilities and other more restrictive forms of care by virtue of its range of facility sizes, residential care helps older people remain in their neighborhoods of residence for longer, supporting them in functional impairment and reducing isolation and loneliness, which have been linked to a number of adverse health conditions (Cornwell & Waite, 2009; Jungers, 2010; Leggett et al., 2011).

As a result, the demand for residential care is expected to grow, putting pressure on cities and local governments to incentivize and plan for more care facilities to meet the growing long-term care need, particularly in the highly populated and urbanized regions of California, the largest state by older adult population. At present, more than a third of residential care facilities are located in the western US (Harris-Kojetin et al., 2013), and more than a quarter of facilities nationwide exist in California, accentuating the importance of long-term care and the residential care industry in the state (California Department of Social Services [CDSS], 2018).

Little is known, however, regarding the number and distribution of residential care facilities in Californian cities and surrounding areas, containing large populations of older adults who need or will soon need such care. The vast majority of facilities in the state are situated near coastal areas, in major metropolitan centers, as demonstrated by Frochen et al. (2019) in evaluating the distribution of residential care in the City of Los Angeles, and not in other major cities in California and their broader geographies (CDSS, 2018). But in the more socially and economically diverse urban cores of Californian cities, access to residential care and other forms of housing varies substantially by age, socioeconomic status, gender, and other demographic variables, all of which influence the location and geography of the housing type (Joint Center for Housing Studies, 2014; Thomas et al., 2018).

Because residential care is regulated primarily at the state level in the US and is funded and developed almost exclusively by private means, it is subjected to less governmental oversight and regional planning than institutional facilities such as skilled nursing care, which have historically been planned and developed via federal partnerships (Moroney & Kurtz, 1975). The development of residential care, in other words, is guided mainly by market forces such as demand, land availability, local planning regulation, and in the case of larger, higher end facilities, the existence of affluent populations that can afford the service, rather than any evaluation of need with respect to the older adult population. The investigation of residential care development, then, is perhaps best conceptualized through the lens of health needs assessments, in which health and long-term care resources are analyzed not just economically (through supply and demand), but from a larger social and population health perspective, considering the needs of older adults and prioritizing supportive housing resources accordingly, especially among older women with functional impairment, the typical demographic of the facility type, representing roughly three quarters of residents in such facilities (Caffrey & Sengupta, 2018; Wright et al., 1998).

Since the residential care industry in California is concentrated in major metropolitan areas, and because the development of such facilities varies substantially by neighborhood, in this study, we present spatial and cartographic analyses of the availability of residential care in the two largest Californian cities and surrounding counties by census tract, using 2015 California Department of Social Services residential care for the elderly and American Community Survey data. To understand the geography of residential care in these areas, we map the ratio of facility beds to older women with a disability. To evaluate the spatial distribution of facilities, we present maps of locations and spatial clustering of small and large facilities in California’s largest cities and residential care markets relative to the concentration of older women with disability.

## 2. Materials and methods

### 2.1. Data

We acquired 2015 California Department of Social Services (CDSS) residential care facility data, a census of all licensed facilities in the state, and geocoded facilities based on their address information. We also used data from the 2015 American Community Survey (ACS) 5-year estimates to create measures of the proportion of older women with disability in the census tract, which serves as a proxy for the potential long-term care need in the older adult population. In ACS, disability is defined as having hearing, cognitive, ambulatory, self-care, or independent living difficulty (Social Explorer, 2018). Geocoded residential care facilities and census tracts were then spatially joined using the North American Datum (NAD) 1983, California State Plane 2011V FIPS 0405 Feet Lambert Conformal Conic projection to explore the relationship between the number of beds and older adults in tracts who may currently or in the future require such care. The spheroid of this datum and projection is the Geodetic Reference System (GRS) 1980 (Esri, 2016). We limited the scope of the analysis to Los Angeles and San Diego Counties, the two largest counties in the state by population, which collectively represent more than one-third of the entire state’s population (U.S. Census Bureau, 2017a, 2017b). The next largest cities in California, San Jose and San Francisco, as part of the San Francisco Bay Area, were excluded from the analysis due to the small residential care presence in either jurisdiction and lack of spatial clustering of facilities compared to Los Angeles and San Diego. Although the Bay Area is an important metropolitan area in California, the residential care industry is considerably limited there, as well as in all of Northern California, compared to Southern California and its largest metro areas.

### 2.2. Analysis

To spatially analyze residential care facility beds and older women with disability, we calculated the number of beds and older women in Los Angeles and San Diego County census tracts. In evaluating the relationship between beds and older women, we mapped the ratio of beds and older women, categorizing the ratio of the two as above and below the adequate supply and undersupply equilibrium break point of 1.0. Densities of beds and older women and the ratio of the two were categorized and illustrated in county choropleth maps, using graduated colors to show increases in values of each in census tracts.

To investigate the spatial distribution of facilities, we employed hot spot analysis, testing for clusters of small and large sized facilities using the Getis-Ord Gi* statistic (Esri, 2018). Applying the method, the size of each facility was compared to others near it, utilizing a distance band such that every facility retains at least one nearest neighbor, displaying statistically significant facilities that are closer and more alike to one another in size than others within the band. The distance band for City of Los Angeles and San Diego facilities were three and one-quarter miles and just over four miles, respectively. We constrained hot spot analyses to the largest and principal cities in both Los Angeles and San Diego Counties, namely, the Cities of Los Angeles and San Diego. Facilities were symbolized in maps with red denoting concentrations of large facilities, blue indicating concentrations of small facilities, and no color indicating statistically insignificant or randomly dispersed facilities in terms of size. The darker the color of red or blue for clusters of large and small facilities, respectively, the greater the statistical significance of the hot spot, ranging from 90% to 95% to 99%, indicating the level of confidence that an extreme sized facility, whether small or large, is located near other extreme sized facilities. They were then illustrated on top of city census tract choropleth maps, illustrating the concentration of older women in each jurisdiction, showing where smaller facilities (board and care homes of six or fewer beds) and larger facilities (assisted living and continuing care retirement communities (CCRC) of 25 beds on average in size) exist relative to densities of the potential long-term care need. In city choropleth maps, areas shown in white, absent of dots indicating no older women, represent unincorporated neighborhoods under county jurisdiction.

## 3. Results

We show the spatial distribution of residential care in Los Angeles County and its principal city, Los Angeles and in San Diego County and its principal city, San Diego.

The main map portrays the ratio of residential care facility beds to older women in Los Angeles County census tracts in Figure ①, highlighting areas with no beds per older women, as shown in white, areas with less than one bed per older woman, as represented by the lightest shade of gray, and areas with more than one bed per older woman, as shown in darker gray and black. Ratios shown in the lightest gray represent areas of undersupply or fewer beds than older women. Ratios shown in darker gray to black represent areas of adequate supply or more beds than older women. The majority of county census tracts show no beds per older women or an undersupply of beds, whereas only select and widely scattered areas of the county indicate an adequate supply. The highest ratios of adequate supply in the county occur in Santa Monica, Claremont, south of Beverly Hills, portions of the San Fernando Valley, Long Beach, Alhambra, Arcadia, south of Torrance, and north of Humphreys and Lancaster, toward the northern end of the county.

The map displays the location and clustering of residential care facilities in the City of Los Angeles in Figure ③, drawn over the concentration of older women with disability. The map shows where facilities are sited, where clusters of small and large facilities congregate, as shown in blue and red respectively, and where large enclaves of older women are located, increasing in density, as shown in dot patterning to gray to black, indicating zero to low to high density. Dot patterning in city census tracts specifies areas with no older women, typically representing open space or public areas, such as Griffith Park and Topanga State Park. Areas shown in white within the larger boundary of the City of Los Angeles denote independently incorporated cities, including San Fernando, Beverly Hills, and Culver City. The majority of facilities are located in the San Fernando Valley, most of them small board and care facilities, six or fewer beds in size. Fewer facilities are located south of this suburban area, inside and west of the downtown district, most of them large assisted living and CCRC facilities. In general, clusters of small facilities group in areas of lower densities of older women, such as the San Fernando Valley, while clusters of large facilities congregate in areas of higher densities of the demographic, surrounding and west of the downtown area. However, certain areas with high concentrations of older women lack an immediate residential care presence, such as portions of Hollywood and neighborhoods east, west, and south of the downtown area.

The map document also shows the spatial distribution of residential care in San Diego County and the City of San Diego in Figure ②. It shows the ratio of residential care facility beds to older women in county census tracts, displaying areas with zero beds per older women, as depicted in white, areas with less than one bed per one older woman, as shown by the lightest shade of gray, and areas with more than one bed per one older woman, as shown in darker gray and black. Similar to Los Angeles, the majority of county census tracts show no beds per older women or an undersupply of beds, whereas only select and fairly dispersed areas of the county indicate adequate supply. The greatest ratios of adequate supply in the county take place in central San Diego, south of La Presa, south of Escondido, and north of Encinitas.

The main map displays the siting and concentration of residential care facilities in the City of San Diego, illustrated on top of the density of older women with disability in Figure ④. It shows the location of facilities, clusters of small and large facilities, and enclaves of older women. As in Los Angeles, the categorization of the density of older women increases from dot patterning to gray to black, representing zero to low to high density. Dot patterning in city tracts indicates areas with no older women, representing military installations, including Miramar. The majority of facilities are located in the southern half of the city, where most of the clustering of small and large facilities arises. Fewer facilities are located in the northernmost portion of the city, where only small amounts of clustering take place. Unlike Los Angeles, clusters of small facilities in San Diego do not congregate in areas of lower densities of older women, and clusters of large facilities do not coalesce in areas of higher densities of the demographic. In fact, a nearly opposite pattern takes place in the map, in which significant groupings of large assisted living and CCRC facilities cluster adjacent to lower densities of older women in La Jolla, and substantial concentrations of small facilities cluster in neighborhoods of higher densities of older women near Lincoln Park. However, in general, facilities in the jurisdiction appear to follow the pattern of densities of older women, with the development of facilities in or near higher densities of older women, as opposed to the lowest density of older women, as shown in the lightest shade of gray.

## 4. Discussion

We show that residential care in California’s two largest counties is largely undersupplied with respect to the potential long-term care need. We also demonstrate that the patterns of residential care facility development and hot spots of small and large facilities in terms of size more or less match the pattern of concentrations of older women with disability, with the exception of the City of San Diego, in which clusters of large facilities are located in neighborhoods with smaller densities of older women and clusters of small facilities are located in neighborhoods with larger densities of older women.

The overwhelming majority of census tracts display no supply of residential care facility beds or an undersupply of beds, emphasizing the limited and exclusive nature of the industry, which faces many institutional and economic obstacles to growth. These obstacles include a shortage of land supply in metro areas, difficulties in facility development permitting where applicable, and a growing but privileged clientele, who pay an average of $3,500 per month for residential care in the US (Joint Center for Housing Studies, 2014). These forces also explain to some extent the odd development patterns seen in cities such as San Diego. In the city, large scale assisted living and CCRC facilities cluster in the affluent La Jolla area, which contain smaller densities of older women who can afford the high-end service, whereas smaller board and care facilities concentrate in the less wealthy and dense Lincoln Park vicinity. In other words, in the relatively spacious and wealthy La Jolla neighborhood, developing residential care is much less complicated than in other areas of the city with greater densities of middle-class older women, where development is almost certainly limited by a lack of both vacant land and a demand for larger, more expensive facilities and where the only practical option is opening smaller facilities in existing homes.

In focusing on areas of adequate supply in Los Angeles and San Diego Counties, a number of scattered census tracts come into high relief. In Los Angeles County, the most extreme examples of adequate supply occur in Santa Monica, Lakewood, Van Nuys, Claremont, and in and near Beverly Hills. Taking a closer look at the underlying data, in each of these tracts, which represent the highest ratios of facility beds to older women as shown in black in the map, there are less than ten older women per square mile, and in one case, as few as two per square mile. In these same tracts, however, there are as many as 80 beds per square mile. In comparison, in census tracts with lower ratios of beds to older women, the number of older women per square mile is typically greater, as is the number of beds per square mile. In other words, the typical configuration of the ratio of beds per older women is greater in both the numerator and denominator, creating a distribution of ratios above .9 that overwhelmingly falls beneath 6.0 and a distribution of ratios below 1.0 that represent the bulk of the dataset, as shown in the map.

In San Diego County, the most extreme instances of adequate supply take place near La Presa, Oceanside, coastal and central San Diego, the eastern outskirts of San Diego, Escondido, and Encinitas. Due to the miniscule square mileage of some of these census tracts, which produce extremely large ratios of facility beds per square mile and older women per square mile, comparing the distribution of total beds and total women is more helpful in analyzing the greatest ratios of beds to older women in the county. The range of total women in these tracts is one to 357, whereas the range of total beds is six to 1334, the smallest and largest of each corresponding to each other. In comparison, the total number of beds appears to grow more stable in tracts with lower ratios of beds to women, and the total number of women seems to increase as well. As with Los Angeles, the vast majority of census tracts contain a ratio of beds to women under 3.4, as illustrated in the map, and a distribution of ratios below 1.0 that represent most of the data.

Areas of adequate supply in both counties appear primarily to be the result of extremely low counts of older women compared to facility beds in census tracts, perhaps indicative of tracts that older women are pushed or pulled to move from to live in other locations (Wiseman, 1980). It is almost certainly the case as well that older women and men without disability or with at-home services and supports live in facilities in such neighborhoods, which would increase the potential need and decrease extreme bed per older adult ratios found in either county. In the only other study investigating the spatial distribution of residential care or any other form of senior housing at the neighborhood level of analysis, Frochen et al. (2019) explain that these areas may also be indicative of communities with planning policies supportive of residential care and other forms of senior housing, such as the Eldercare Facility Ordinance of Los Angeles. Although it is difficult to estimate ratios of beds to older women or older adults that appropriately gauge the current need for residential care, areas of undersupply exemplify neighborhoods that could benefit from such planning policies given the large older adult population, whereas areas of adequate supply illustrate places that more than likely possess such senior housing policies or do not need them due to sheer demand and more affluent populations in support of the industry.

Accordingly, understanding the spatial distribution of residential care can help city and regional planners, community stakeholders, and local representatives address areas of concentrated and underserviced long-term care need through administrative and discretionary planning tools that encourage development of this critically important type of senior housing. In other words, research of this kind, on the geographical disparities in healthcare, can help governmental agencies and communities pinpoint and address the long-term care need of older adults with respect to residential care supply and other forms of housing services and supports outside of the home.

Related to counts of older women in tracts are some of the limitations of this research, including 2015 ACS census tract estimates. 2015 ACS estimates were based on sampling over 60 months of time and are not a complete enumeration of people, as in the 2010 Decennial Census (U.S. Census Bureau, 2018). Although they are more reliable than one-year or three-year estimates, they are still subject to error, particularly when measuring distinct populations such as older women with at least one disability in Los Angeles and San Diego Counties. Further, the ACS measure of disability accounts for various forms of functional impairment, including ambulatory, self-care, and independent living difficulty, but also vision and cognitive impairment (Sex by age by disability, 2015), two forms of limitation that may not necessarily predict the need for residential care or may require more attentive care such as skilled nursing.

Future research is needed to track the increase of residential care in Los Angeles and San Diego along with other major cities in California, investigating how residential care has increased by number of facilities and total beds in jurisdictions relative to the potential long-term care need of older adults through time series analysis of residential care and older age population growth.

## 5. Geolocation Information

In decimal degrees, the geographical coordinates of the City and County of Los Angeles are 34.052235, −118.243683 (WGS84 datum). The geographical coordinates of the City and County of San Diego are 32.715736, −117.161087 (WGS84 datum) in decimal degrees.

### Software

ArcMap as part of Esri’s ArcGIS was used to produce all of the maps in this article. California Department of Social Services (CDSS) residential care facilities were geocoded and spatially joined with Los Angeles and San Diego County census tracts, and spatially referenced using the North American Datum (NAD) 1983, California State Plane 2011V FIPS 0405 Feet Lambert Conformal Conic projection. The spheroid of this datum and projection is the Geodetic Reference System (GRS) 1980. We symbolized the data in graduated color choropleth maps, using the Jenks Natural Breaks Classification Method, which maximizes the variance between classifications, while minimizing the variance within each classification. The only exception to this is the variable of the ratio of beds to older women in either county, in which we distinguished classifications as either above or below the equilibrium point of 1.0, between the second and third classifications, to categorize census tracts as having either oversupply or undersupply of residential care. Finally, we plotted residential care facilities, including clusters of small and large facilities, in the Cities of Los Angeles and San Diego, on top of choropleth maps of older women with disability, showing where concentrations of small board and care and large assisted living and continuing care retirement communities (CCRC) occur relative to the potential need in either jurisdiction.

## Supplementary Material

Main Map
