# Supplementary material for: Residential care in California
Source: J Maps. Author manuscript; Available in PMC 2020 Oct 21. (PMC7577399; doi:10.1080/17445647.2020.1768446)
Supplement: Main Map [file NIHMS1603217-supplement-Main_Map.pdf]

Residential Care in California

Stephen Frochen[1], Seva Rodnyansky, Ph.D.[2], and Jennifer Ailshire, Ph.D.[1]
[1]USC Leonard Davis School of Gerontology,[2]UC Berkeley Goldman School of Public Policy

Projected Coordinate System: California State Plane 2011 V FIPS 0405 Feet
Projection: Lambert Conformal Conic
Geographical Coordinate System: NAD83

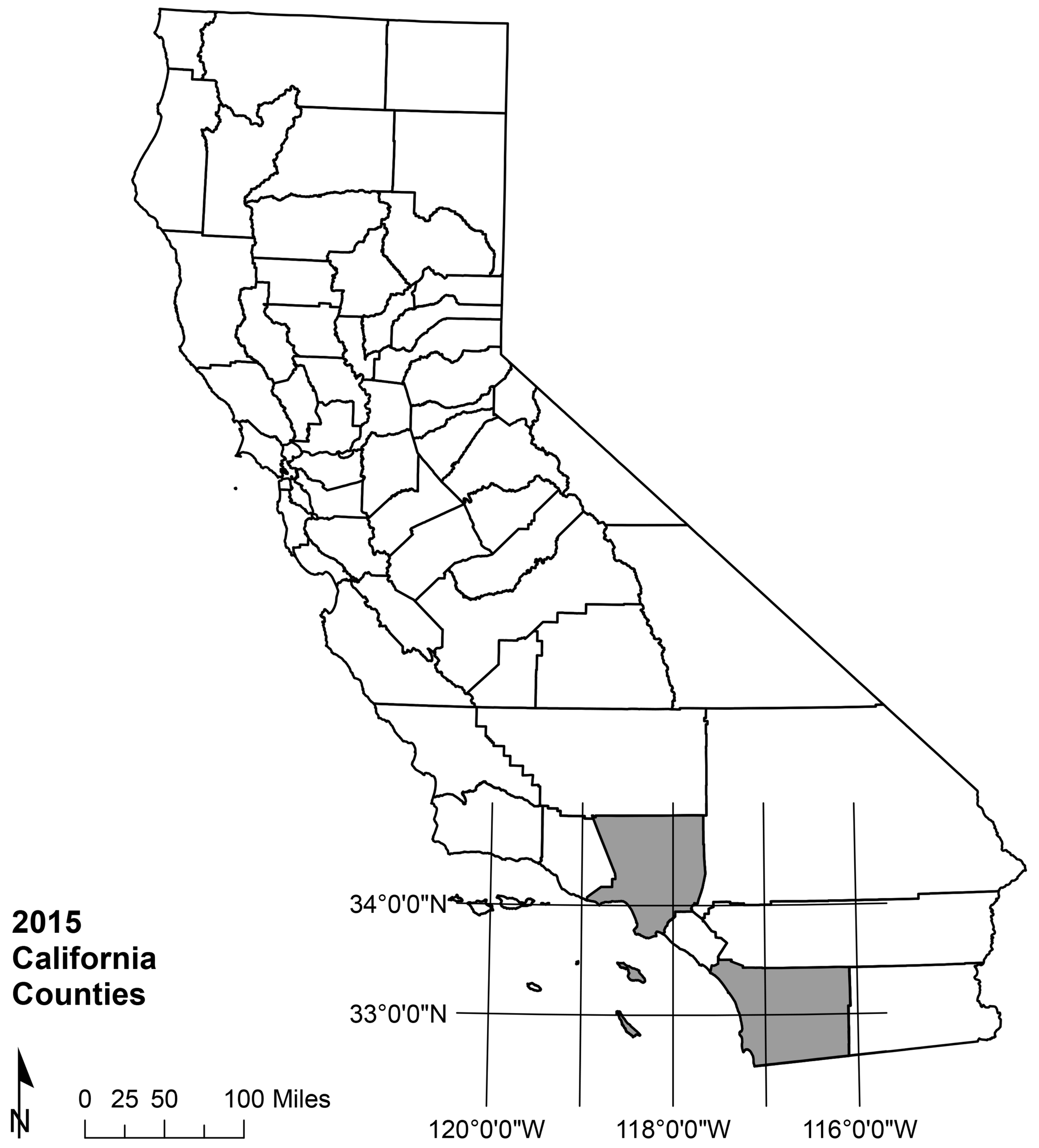

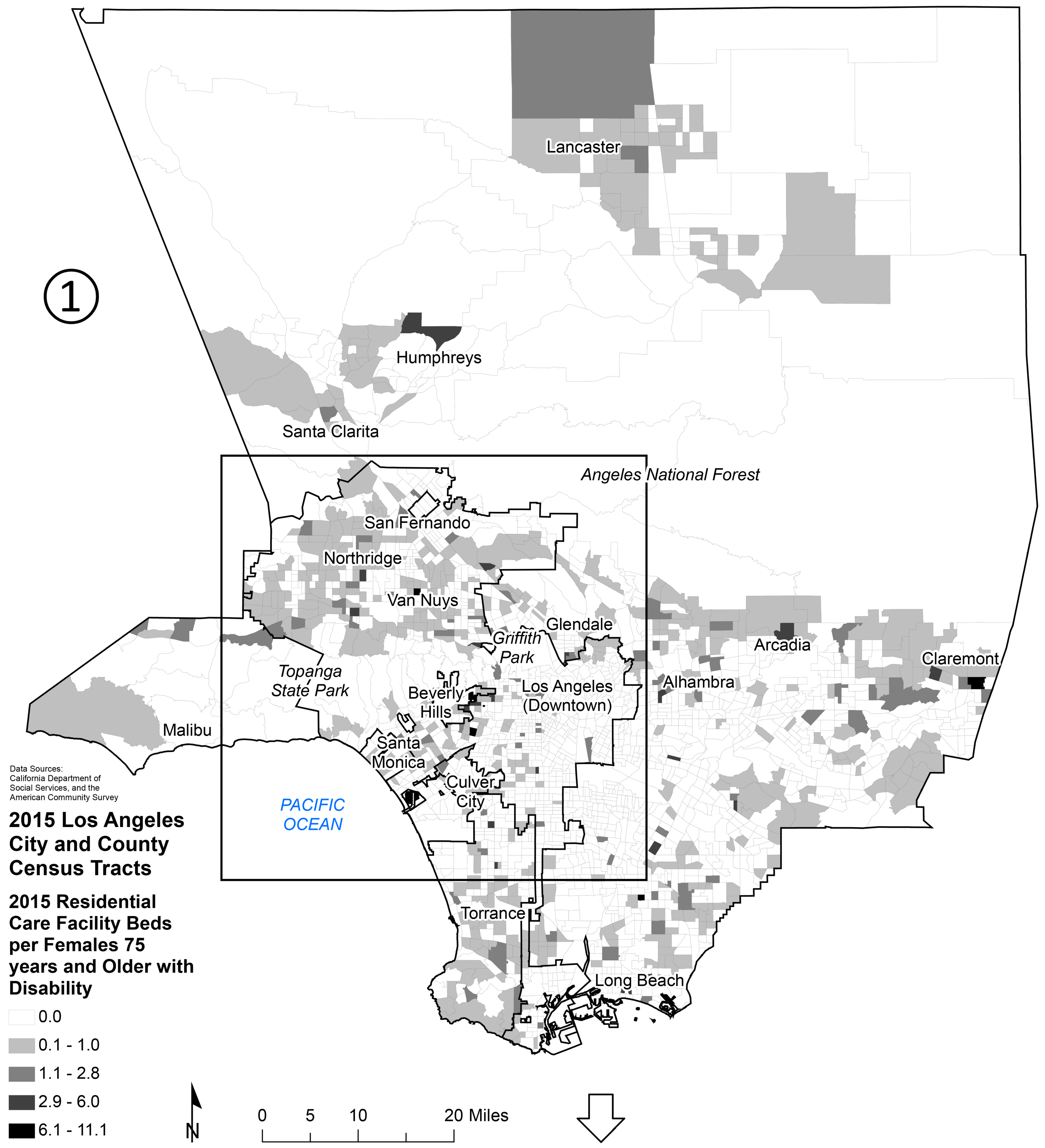

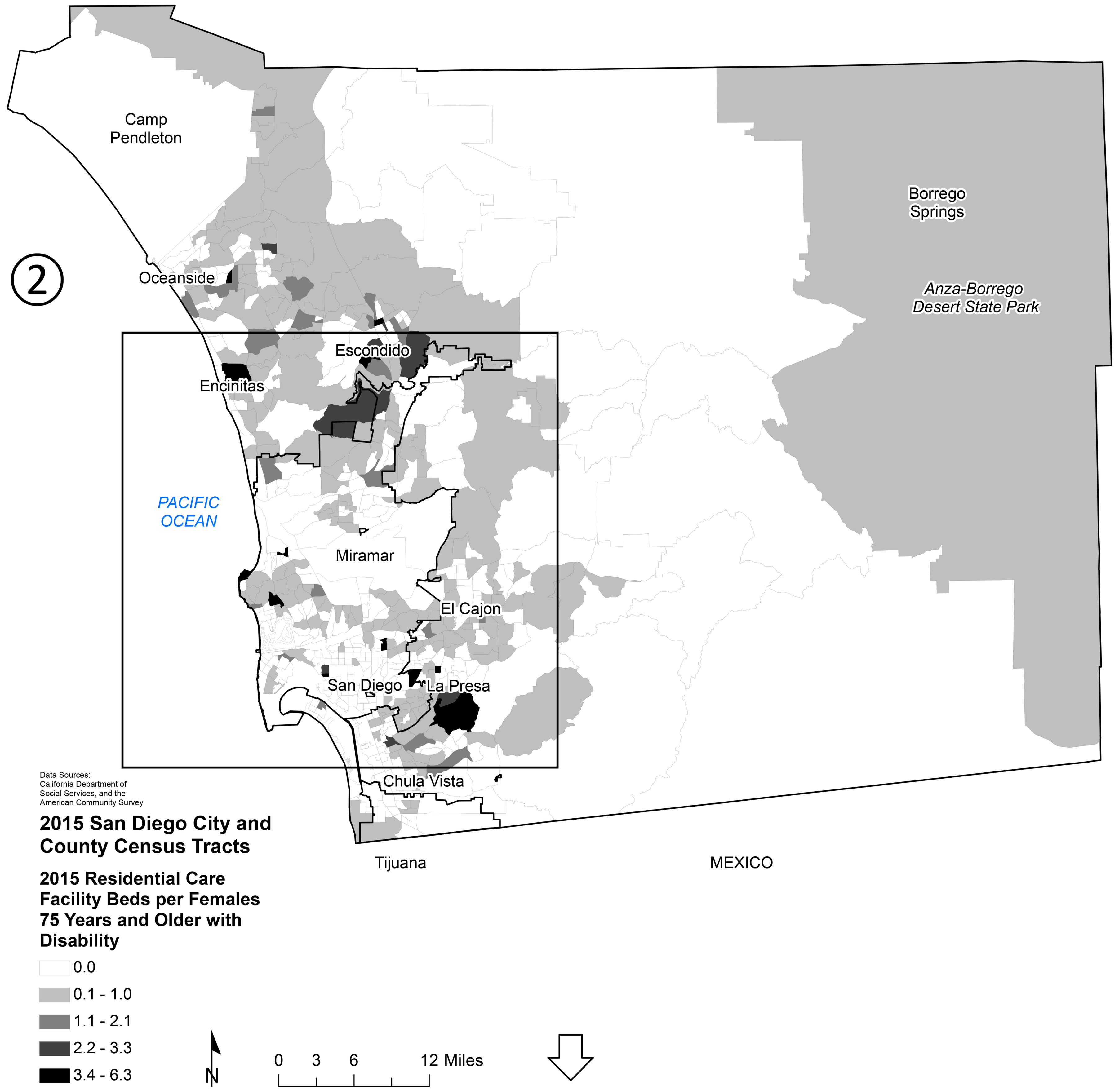

Residential Care in California

As life expectancy increases in the U.S., the number of years spent in disability is also increasing, requiring expansion of non-institutional long-term care for functionally impaired older adults in the absence of adequate formal and informal care in the home. Residential care, the fastest growing and largest type of housing for the elderly by number of facilities, bridges the gap between at-home and institutional care for older adults with disability, and the Western United States, primarily California, contains the most facilities. Although residential care is growing as an industry and is increasing in concentration in metropolitan areas in the state, it is unequally distributed in cities and is undersupplied relative to the potential long-term care need of older disabled people.

Objectives and Methods: We examined the availability of residential care in Californian cities, showing geographical disparities in the ratio of care supply and need. 2015 California State Department of Social Services residential care facility data were linked with 2015 California American Community Survey (ACS) data to analyze the geographical distribution of residential care compared to the prevalence of old age disability.

① ② We calculated the number of residential care beds per square mile, the number of older women (75+) with at least one disability (the typical residential care demographic) per square mile, and mapped the ratio of beds to older women with disability in Los Angeles and San Diego County census tracts. We focused on Los Angeles and San Diego Counties, as they represent the largest markets for residential care in the state and a combined population a third the size of California.

③ ④ We also mapped clusters of small and large facilities relative to the care need in the Cities of Los Angeles and San Diego to show geographical trends in the development of small board and care facilities and large assisted living facilities or continuing care retirement communities.

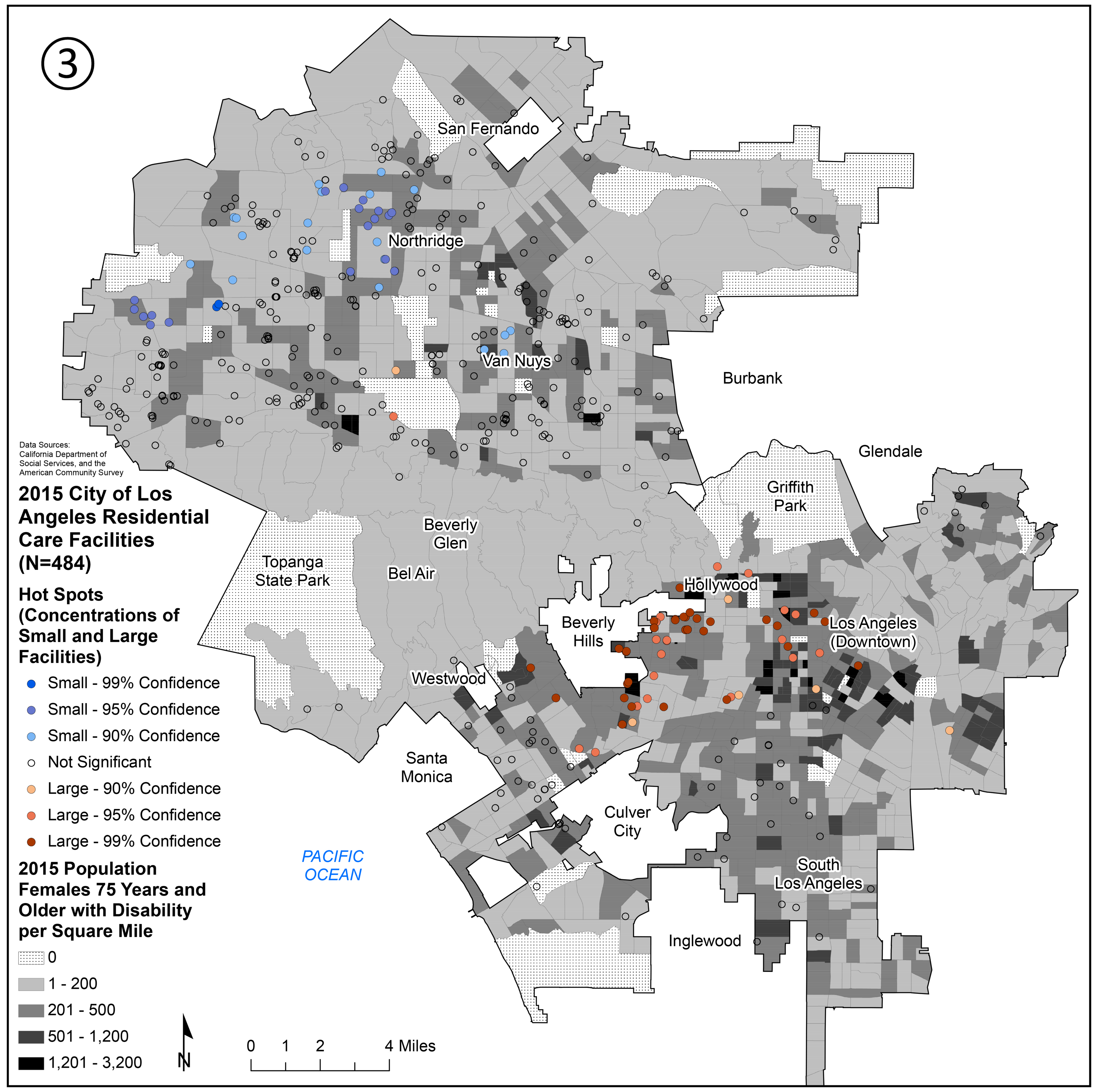

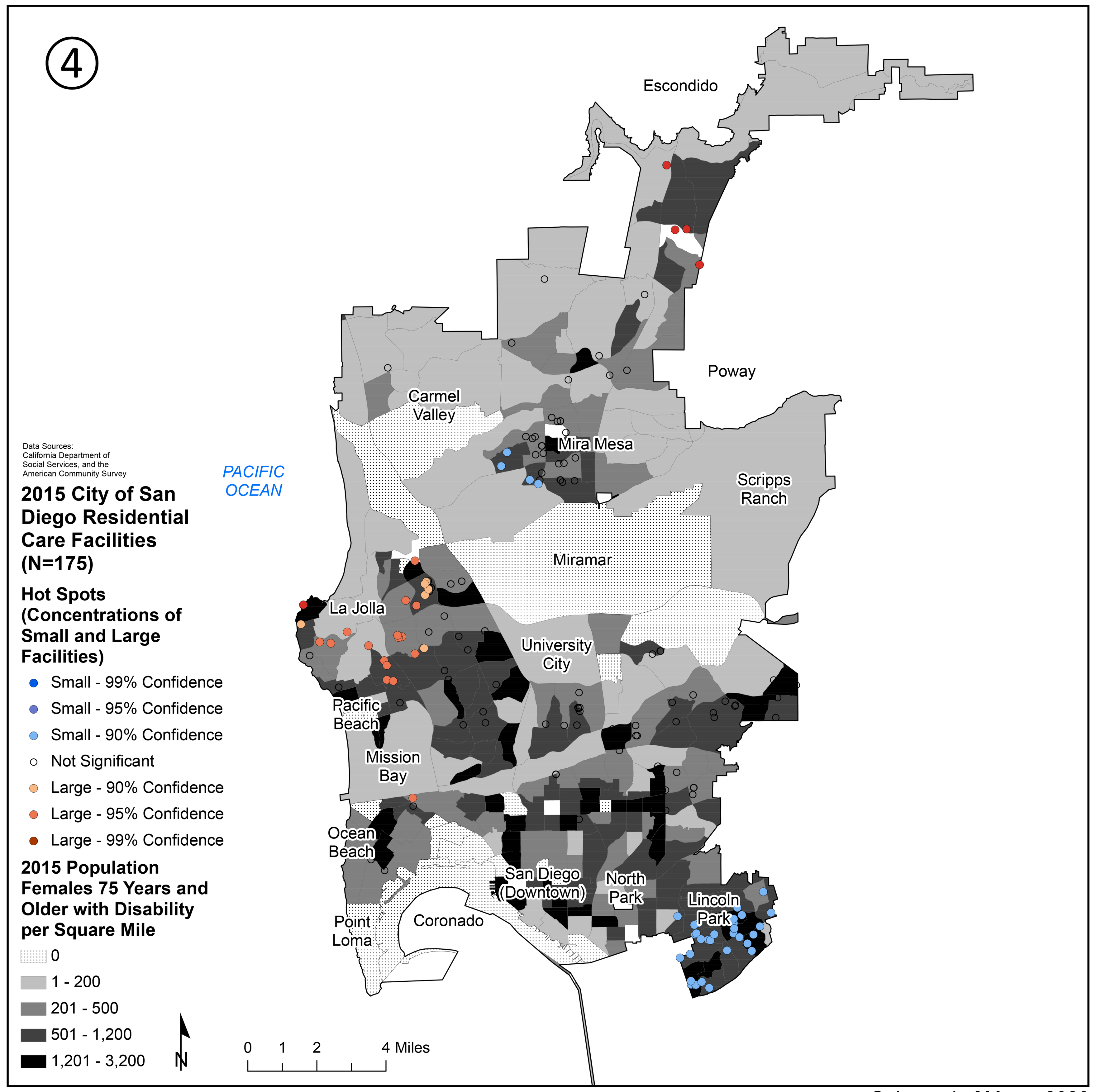

© Journal of Maps, 2020
